# Factors which influence ethnic minority women’s participation in maternity research: A systematic review of quantitative and qualitative studies

**DOI:** 10.1371/journal.pone.0282088

**Published:** 2023-02-24

**Authors:** Holly Lovell, Sergio A. Silverio, Lisa Story, Emily Skelton, Jacqueline Matthew

**Affiliations:** 1 Maternity Services, Guy’s and St Thomas’ NHS Foundation Trust, London, United Kingdom; 2 Department of Women & Children’s Health, School of Life Course & Population Sciences, King’s College London, London, United Kingdom; 3 Division of Radiography and Midwifery, City University of London, London, United Kingdom; 4 Department of Perinatal Imaging and Health, School of Biomedical Engineering & Imaging Sciences, King’s College London, London, United Kingdom; University College London, UNITED KINGDOM

## Abstract

**Background:**

Women from Black, Asian and mixed ethnicity backgrounds in the UK experience higher rates of maternal and neonatal mortality and morbidity, and report poorer experiences of maternity care. Research is required to understand how to reduce these disparities, however, it is acknowledged these groups of women are under-represented in clinical research.

**Aim:**

To investigate factors which influence participation in maternity research for women from an ethnic minority background.

**Methods:**

A systematic review was conducted to examine influencing factors for research participation. MEDLINE/CINHAL/PsycInfo/EMBASE databases were systematically searched in March 2021 and updated in March 2022. Papers were eligible if they explored maternal research participation and identified a woman’s ethnicity in the results. No restrictions were placed on methodology. A convergent integrated approach was used to synthesise findings.

**Findings:**

A total of 14 papers met the inclusion criteria. Results were divided into eight overarching themes. A personalised approach to recruitment and incorporating culturally sensitive communication and considerations enhanced research participation. Distrust around sharing data, a perception of risk to research participation, and research lacking in personal relevance adversely affected the decision to participate. Large variation existed in the quality of the studies reviewed.

**Conclusions:**

Consideration of a woman’s culture and background in the design and the delivery of a maternity research study may facilitate participation, particularly when sampling from a specific population. Further research, informed by women from ethnic minority backgrounds is warranted to develop women-centred recommendations for conducting inclusive maternity research.

**Prospero registration:** www.crd.york.ac.uk/PROSPERO/display_record.asp?ID=CRD42021261686.

## Introduction

### Ethnic inequalities in maternity care

In the United Kingdom (UK) women from Black, Asian, or ethnic minority backgrounds experience worse maternal morbidity and mortality than their White counterparts. The most recent ‘Mothers and Babies: Reducing Risk through Audits and Confidential Enquiries across the UK’ (MBRRACE-UK) report into maternal deaths during pregnancy and the first six weeks postnatally found Black women’s mortality rate is quadruple that of White women. Women of mixed ethnicity are more than three times more likely to die, and Asian women are almost twice as likely to die compared to White women [1]. Additionally, Black women are twice as likely to have a stillbirth and neonatal mortality is 43% higher compared to the White population. Asian women are around 60% more likely to suffer a stillbirth or neonatal death [2].

Maternal mortality is just one indicator of existing disparities and does not represent the multiple inequalities women from Black, Asian and minority ethnic groups experience [3]. Ethnic minority women have an increased risk of premature birth [4], gestational diabetes [5], pre-eclampsia [6] and are more likely to have poorer mental health experiences, yet are less likely to be offered support [7, 8]. Other disparities likely exist, however as no UK mandatory reporting system exists for near misses and serious morbidity in maternity additional inequalities may be concealed [9].

There are several perceived causes for these racial disparities and intersectionality is likely key; some women from ethnic minorities experience multiple disadvantages. MBBRACE-UK found 90% of women who died had multiple problems, including living in more deprived areas, physical and mental co-morbidities, and being obese or overweight [10].

Ethnic disparities are often attributed to lower socio-economic background statuses. Although this may be a factor for some, increasing evidence suggests women from ethnic minority backgrounds who are not classified as disadvantaged continue to experience poorer outcomes [4, 11, 12]. Potential additional causes to be considered include inadequate maternity staff training in how to meet differing cultural needs [13, 14] and experiences of racism and discrimination from healthcare staff, resulting in concerns not being addressed or heard [15–17].

Racial disparities within maternity are not new, yet a paucity of high-quality research is available to inform care improvements for this population [18].

### Under-representation of ethnic minority groups in clinical research

There are calls for research to reduce ethnic disparities in maternity care [11, 15, 19]. Yet existing clinical research includes an under-representation of people from Black, Asian and ethnic minority backgrounds [20]. Currently no published data exists on whether participants in maternity research represent the different ethnic groups accessing maternity care. This may partly be due to the lack of national maternity research databases, along with poor practices in collecting and reporting ethnicity data [21] and no agreed standard for reporting ethnicity in journals However, it is well acknowledged in other specialities that participants do not reflect the ethnic diversity of the populations from which they are recruited [22–25]. One UK survey demonstrated people who identified as White British were 87% more likely to have taken part in research compared to those from Black, Asian and minority ethnic backgrounds [26]. Findings from research which is not representative of populations may have reduce generalisability [27] and differences in efficacy of interventions may be missed [28].

Improving diversity in clinical research is identified as a key priority; a recent report from the Commission on Race and Ethnic Disparities recommended barriers and solutions to research participation by ethnic minorities should be investigated [29]. Additionally, “what are the best approaches to ensure inclusion and participation of under-represented and vulnerable groups in clinical trials” has been identified as one of 10 research priorities to be investigated to improve trial recruitment [30].

Training and toolkits were developed to improve an inclusive approach to research design [20, 31]. However, they are not specific to maternity and tailored approaches are recommended for specialist areas. Maternity research poses additional difficulties; a time sensitive nature of eligibility with narrow gestational windows. Furthermore, when deciding whether to participate women consider potential risks both for themselves and their unborn child.

To improve the representation of ethnically diverse populations in clinical trials, we must first understand factors influencing participation [32]. This paper aims to systematically review papers that investigate factors influencing participation in maternity research for women from ethnic minority backgrounds.

## Methods

The protocol review is registered on PROSPERO: www.crd.york.ac.uk/PROSPERO/display_record.asp?ID=CRD42021261686.

A previous systematic review of pregnant women’s participation in research was used to initially identify search terms [33]. A Population Exposure Outcome (PEO) analysis of the research question identified additional search terms (Table 1), particularly those related to ethnicity.

**Table 1:** PEO analysis of search terms.

Population		Population		Exposure		Exposure		Outcome
pregn* OR expecting woma* OR antenatal OR prenatal OR exp Pregnancy (MeSH) OR exp Maternal Health Services/ OR exp Prenatal Care/ OR exp Midwifery (MeSH)	**A** **N** **D**	ethnic* OR black OR Asian OR minority OR BAME OR exp Ethnic Groups (MeSH).	**A** **N** **D**	participat* OR enrol* OR include* OR recruit* AND	**A** **N** **D**	stud* OR trial* OR research OR exp Research (MeSH)	**A** **N** **D**	challeng* OR reason* OR motivation* OR view OR decision* OR attitude* OR willing* OR consider* OR concern* OR barrier* OR issue

A systematic search was undertaken of MEDLINE/CINHAL/PsychInfo/EMBASE in March 2021, to identify relevant articles, updated in March 2022. The initial MEDLINE search strategy can be seen in S1 File. Keyword searches were undertaken using truncation functions to increase the search breadth, alongside relevant MeSH subject headings; no limits were applied. Additional papers were identified through reference lists of relevant studies.

Reviewer one (HL) reviewed titles to exclude obvious non-related papers and screened abstracts to exclude those not meeting the inclusion criteria. Reviewer two (JM) assessed a randomly selected 25% of abstracts after the title screen to check for agreement. Where it was unclear whether the paper met the inclusion criteria, it was retrieved for full text review and consensus agreement by the two reviewers. The remaining papers were retrieved for full text review and each author independently assessed whether the inclusion criteria was met (Table 2). In the case of any disagreement, discussion was undertaken to examine papers together until consensus was achieved.

**Table 2:** Inclusion/exclusion criteria.

Inclusion Criteria	Exclusion Criteria
Original research of any design, including quantitative, qualitative, or mixed methods studies OR Secondary analysis of primary research	Reviews, opinion paper, journal letters, conference proceedings
Aims include examination of factors that affect decision to participate in research	Descriptions of recruitment strategies which do not include analysis
Population includes extractable data from women who were recruited during pregnancy	No clear identification of different ethnic groups in the results
Results stratified by ethnicity are extractable	
Access to the full text paper	
Paper available in the English language	

### Data extraction and synthesis

HL extracted data to capture the papers main characteristics, which was checked by JM (Table 3).

**Table 3:** Summary of study characteristics.

Author (year)	Original study design	Methods of original study	Country	Study Design—secondary article	Aim—secondary paper	Methods—secondary paper	Study sample secondary article	Participants ethnicity and migrant status*
Mallet et al (2020)	Randomised controlled trial (RCT)	Induction of labour for low risk primiparous women between 39–39+4 versus expectant management.	USA	Quantitative descriptive	To evaluate maternal characteristics associated with consent to a RCT of labour induction.	Reasons for decline analysed from screening logs.	Pregnant woman between 34-38/40 screened for the RCT (n = 7112) recruited across 33 hospitals.	1154 (16%) Black; 545 (20%) 1405 (20%) Hispanic; 454 (6%) Asian; 3764 (53%) White; 335 (5%) Other/unknown or more than one race
Garg et al (2016)	Hypothetical—Cohort study	Collection of maternal and infant biological samples and information.	UK	Qualitative	To seek the views of people from diverse ethnic backgrounds about participation in a proposed birth cohort.	8 Focus groups organised into women from same, self-reported ethnic background.	Women who were either pregnant or had a child under the age of 5 (n = 40)	8 (20%) White British, 4 (10%) Black British, 6 (15%) African and Caribbean, 7 (18%) Bangladeshi, 4 (10%) Turkish, 5 (13%) Chinese, 6 (15%) Jewish. 29 (73%) were born outside the UK.
Neelotpol et al (2016)	Quantitative Cross-sectional study	Questionnaire and collection of maternal blood, cord blood and meconium for biochemical analysis	UK	Quantitative Descriptive / Analysis of methods	To record and explore influential factors and to devise effective methods of participant recruitment, retention, and sample collection.	Descriptive analysis of recruitment and retention methods, and reasons for decline.	Pregnant women who were approached to participate in the original study (n = 244). 19% (n = 47) declined participation to the original study.	Study group (n = 98): 11 (11%) Bangladeshi, 27 (28%) Indian, 60 (61%) Pakistani origin. 37.8% were British born. Comparison group: 38 (100%) White, 10.5% born outside UK.
Lindsay et al (2021)	Multiple studies	Interviews, surveys, focus groups	USA	Mixed Methods	To present effective recruitment strategies and lessons learnt from recruiting Brazilian immigrants living in the US.	Recruitment logs analysed using descriptive statistics. Thematic analysis of notes, research memos, and progress reports.	Six studies including 233 participants. 2/6 studies included pregnant women first baby (n = 105).	105 (100%) Brazilian (ethnicity not stated), who had been living in the USA for a least 6 months
Brown et al (2015)	Randomised controlled trial	Lifestyle intervention versus usual care, for pregnancies complicated by gestational diabetes or impaired glucose tolerance.	USA	Randomised controlled trial	To assess whether targeted recruitment letters with information specific to an individual’s ethnicity, in their first language, would improve screening and enrolment.	Eligible women were randomly assigned to receive targeted or standard recruitment letters, stratified by medical facility and ethnicity (White vs non-White).	Pregnant women (n = 445)	18 (4%) African American, 252 (57%) Asian/Pacific Islander, 99 (22%) Latina, 86 (19%) White
van Delft et al (2013)	Prospective longitudinal study	Questionnaire and internal USS to assess bladder bowel and vaginal function and symptoms. Visits at 36/40, 3/7PN, 3/12 and 1 year PN	UK	Quantitative descriptive	To identify factors that could influence recruitment in a prospective longitudinal study involving pregnant women.	Reasons for decline were analysed using descriptive statistics, with reasons divided by ethnicity.	Pregnant women (n = 1043)	458 (44%) White, 207 (20%) Asian, 28 (3%) Mixed ethnicity, 218 (21%) Black, 132 (13%) Other.
Barnett et al (2012)	Two RCTs	Women randomised to different breastfeeding support to examine interventions to improve breastfeeding rates and duration. Telephone interviews at 1,3 and 6 months postpartum.	USA	Mixed Methods	To presents the strategies employed to achieve the recruitment and retention rates.	Descriptive analysis of recruitment and retention rates, and recruitment strategies. 20% of participants had a qualitative exit interview at 6 months postnatal.	Women were recruited when pregnant (n = 907) to participate when postnatal.	54 (6%) Non-Hispanic White, 288 (30%) Non-Hispanic Black, 595 (55%) Hispanic, 66 (7%) Asian/Other.
Nechuta et al (2012)	Hypothetical	Collection of biological samples, including maternal blood, cord blood and placenta	USA	Cross-sectional Survey	To evaluate attitudes towards collection and storage of biological specimens, and to assess whether attitudes differed by maternal characteristics	Face to face structured interviews	Pregnant women (n = 311).	180 (59%) Non-Hispanic White, 61 (20%) Hispanic, 51 (17%) Non-Hispanic Black, 14 (4%) Non-Hispanic other.
Nechuta et al (2009)	Hypothetical—Mixed studies	(1) 45 min in-person interview during a clinic visit; (2) 15 min telephone interview antenatally; (3) maternal or infant medical record abstraction; (4) infant physical examination;	USA	Cross-sectional Survey	To assess attitudes towards different data collection procedures, and to associate maternal factors associated with willingness to participate.	Face to face structured interviews	Pregnant women (n = 311).	180 (59%) Non-Hispanic White, 61 (20%) Hispanic, 51 (17%) Non-Hispanic Black, 14 (4%) Non-Hispanic other.
Lamvu et al (2005)	Prospective cohort study	Women were recruited at <12/40 to assess the effects of drinking water disinfection by-products on spontaneous abortion.	USA	Cross-sectional survey	To determine if primary reason for participation varied by race, independent of other factors	Structured telephone interviews at 27 weeks to assess motivation for study participation.	Pregnant women (n = 1106)	735 (66%) White, 285 (26%) Black, 30 (3%) Hispanic, 56 (5%) Other.
Savich et al (2020)	Hypothetical—Birth cohort study	Collection of biological samples for future genetic testing.	USA	Cross-sectional mixed methods	To assess attitudes beliefs and concerns related to biobanking for genetic material. To identify factors contributing to willingness to participate in long-term paediatric studies.	Focus groups and questionnaires.	Women (n = 37) who were either currently pregnant (n = 10), had children (n = 13), or never been pregnant (n = 14)	17 (50%) Hispanic, 14 (38%) White Non-Hispanic (n = 14), 6 (16%) Native American.
Gatny and Axinn (2011)	Cross-sectional survey	Interviews	USA		To examine reasons for participation in pregnancy outcomes research, and whether these differed by race.		Pregnant women (n = 90) however only 87 participants included in analysis.	47 (54%)African American 40 (46%) White
Martin et al (2013)	Randomised controlled trial	To assess the impact of a behavioural education intervention on reducing postpartum depression.	USA	Mixed Methods / Description of methods	To describe a feedback- responsive recruitment strategy, including recruitment rates and reason for decline.	Taxonomy of reasons for decline were analysed weekly to inform adaptations to the recruitment message.	Postnatal women (n = 540 enrolled/128 declined).	205 (38%) Black/African American, 335 (62%) Hispanic/Latina.
Gillespie (2022)	Prospective Cohort Study	To assess the effect of perinatal psycho-neuroimmunology on determinants of spontaneous birth timing	USA	Quantitative descriptive	To compare the success of different recruitment methods	Analysis to compare the effectiveness of the two different recruitment methods.	Pregnant women (n = 96)	96 (100%) Non-Hispanic Black

* Only data from ethnic minority participants were extracted for the review.

Inclusion criteria was not limited to a particular methodology to facilitate a rich understanding of research participation, therefore included studies used quantitative, qualitative, and mixed-methods designs. The quantitative studies mainly employed descriptive statistics which were heterogenous in outcomes measured and measurements used, therefore a meta-analysis was not possible. Where papers included non-minority White participants, only the data relating to ethnic minority participants was extracted for this review.

Data were synthesised using a convergent integrated approach [34]. As each study design were able to answer the research questions, integration of data was performed and separate analysis was not warranted [35].

The convergent integrated approach requires data to be transformed into a compatible format [34]. To answer our research questions we aimed to explore themes; consequently transforming each study into qualitative data was considered most appropriate. Assigning codes or themes to quantitative data is less likely to produce errors compared to assigning a numerical value to qualitative data [36].

Once extracted data were qualitised, data-based convergent synthesis was performed, whereby all data was analysed using the same approach and results were not separated by research methodology [37]. Thematic synthesis was undertaken following Thomas and Harden’s approach [38]. Initial data coding was undertaken independently by HL and JM using NVivo software [39], and files were combined to assess agreement. Discrepancies were discussed until agreement was reached. HL led the development of themes using an inductive approach, which were validated by JM [40]. Initial descriptive themes were presented to a perinatal patient and public advisory group, which explored opinions on the decisions made to date and enabled discussion, facilitating further analytic theme development.

### Quality assessment

The Mixed Methods Appraisal Tool (MMAT) was used to assess quality [41]. The MMAT was chosen as unlike other tools it includes assessment criteria for both mixed methods studies and quantitative descriptive studies, i.e. surveys. Two reviewers independently assessed the papers and discussed discrepancies to reach a consensus. Each study was assessed against five criteria to appraise methodological quality. Responses are categorised as ‘yes’, ‘no’, or ‘can’t tell’. As recommended, papers were not excluded based on quality [41], but the overall standard of papers and risk of bias is considered in the analysis. Studies were considered to be of low methodological quality if they met 0–2 of the criteria, medium quality if they met 3–4 criteria, and high quality if they met all five criteria.

### Patient and public involvement

This systematic review is a component of a larger project, guided by a Community Advisory Group. The group were involved in providing feedback on the research questions and on initial descriptive themes and codes. One of the members reflected on the idea of research participation as personal risk without gain as “what’s the point?”, aptly capturing the concept of risk and perception of research and thus was used as a theme.

## Results

**Fig 1:**
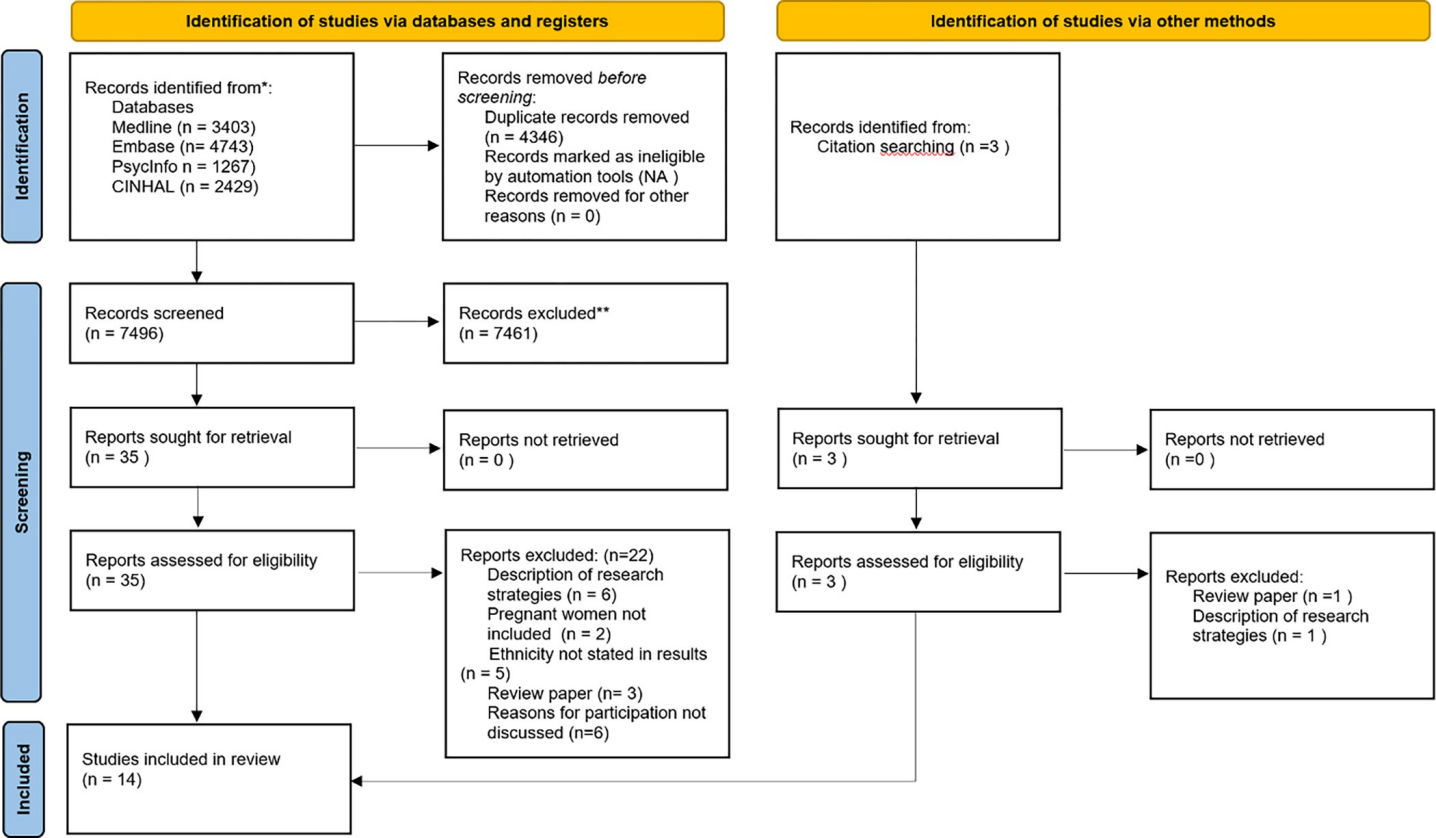
PRIMSA flowchart.

Results are presented as per PRISMA guidelines for reporting systematic reviews [42]. Fig 1 demonstrates the paper selection process. Of the 7496 records screened, 38 papers were retrieved for full text review; fourteen met the inclusion criteria.

### Study characteristics

The summary of study characteristics are presented in Table 3. The majority of studies used either a quantitative descriptive (n = 8) or mixed methods approach (n = 4). One study was a randomised control trial (RCT) and one employed qualitative methodology. Most of the studies were conducted in the USA (n = 11), with three in the UK.

The papers included a variety of approaches to explore research participation. Four were designed to explore women’s opinions on a hypothetical study, and one assessed reasons to participate in pregnancy research in general.

Seven studies were secondary articles investigating factors influencing participation in an original trial. Of these, five focused on analysis of the recruitment methods used in the original trial. One paper tested an intervention to improve recruitment.

Table 4 illustrates the main findings of each paper, based on initial descriptive coding alongside the quality assessment. Only three studies were of high quality, and therefore a low risk of bias. The majority were assessed to be medium quality, however four were considered low quality. This was often due to not being able to accurately determine whether the MMAT quality criteria were met. The full assessment is in S2 File.

**Table 4:** Paper’s findings and quality assessment.

Author (year)	Ethnic minority group data extracted (n)	Reasons identified for participating	Reasons identified for not participating	Quality (MMAT assessment)
Mallet et al (2020)	Black, Hispanic, Asian (n = 3104)		Family influence, preference of usual care	Medium
Garg et al (2016)	Black British, African and Caribbean, Bangladeshi, Turkish, Chinese, Jewish. (n = 32)	Convenience, non-invasive tests, altruism, material incentive, perceived healthcare benefit	Cultural or religious beliefs, concerns about data misuse, distrust, lack of time, invasive tests, perception of risk, lack of benefit	High
Neelotpol et al (2016)	Bangladeshi, Indian, Pakistani. (n = 98)	Cultural understanding, identity of research staff, language resources, participant contact methods, in-person recruitment methods, building rapport, trust in researchers	Cultural or religious beliefs, family influence, language barrier, lack of time, additional burden, lack of interest	Medium
Lindsay et al (2021)	Brazilian (n = 105)	Cultural understanding, identity of research staff, language resources, participant contact methods, convenience, flexibility, material incentive, personal relevance, building rapport, community outreach, positive research experiences, trust in researchers	Concerns about data misuse	Low
Brown et al (2015)	African American, Asian/Pacific Islander, Latina (n = 359)	Language resources, personal relevance		Medium
van Delft et al (2013)	Asian, Mixed, Black, Other (n = 585)		Family influence, language barrier, lack of time, invasive tests	High
Barnett et al (2012)	Non-Hispanic Black, Hispanic, Asian/Other. (n = 853)	Cultural understanding, identity of research staff, language resources, participant contact methods, convenience, flexibility, in-person recruitment methods, material incentive, perceived healthcare benefit, educational benefit, building rapport, community involvement, positive research experiences, trust in researchers	Recruitment approach	Medium
Nechuta et al (2012)	Non Hispanic Black, Hispanic, Non-Hispanic other (n = 126)	Material incentive	Invasive tests	Low
Nechuta et al (2009)	Non Hispanic Black, Hispanic, Non-Hispanic other (n = 126)	Material incentive	Sharing medical records	Medium
Lamvu et al (2005)	Black, Hispanic, Other (n = 371)	Material incentive, perceived healthcare benefit, altrusim		Medium
Savich et al (2020)	Hispanic, Native American (n = 23)	Altruism, personal relevance, trust in researchers, private healthcare insurance	concerns about data misuse, invasive tests, perception of risk	Low
Gatny and Axinn (2011)	African American (n = 47)	Altruism, material incentive, personal relevance	Receiving public healthcare	Medium
Martin et al (2013)	Black/ African American, Hispanic/Latina (n = 540)	Convenience, flexibility, personal relevance, trust in researchers	concerns about data misuse, distrust, lack of time, additional burden, perception of risk, lack of benefit, lack of interest	Low
Gillespie et al (2021)	Non-Hispanic Black (n = 96)	In-person recruitment methods	Recruitment approach	High

### Thematic synthesis

**Fig 2:**
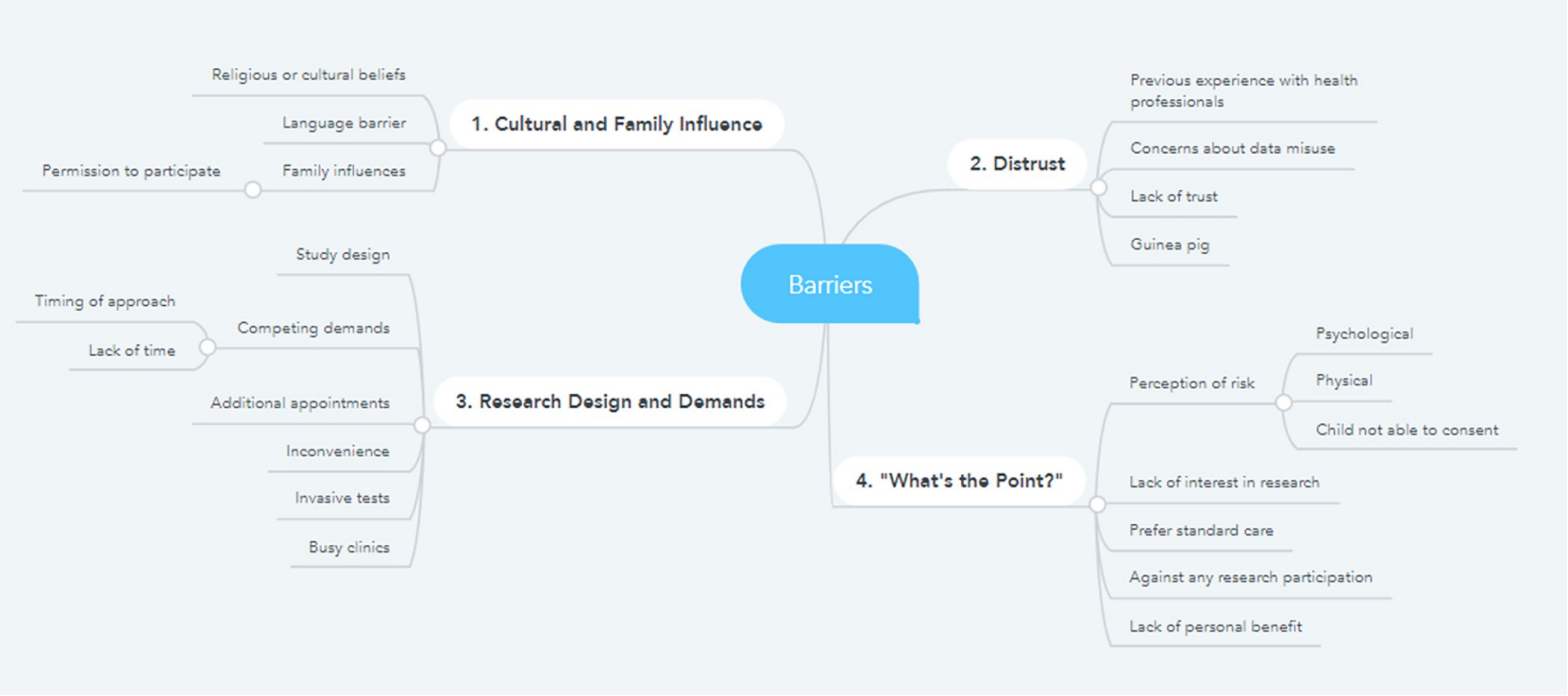
Thematic maps of barriers and facilitators.

Initial coding was divided between factors which were either barriers or facilitators. These were developed into eight themes as demonstrated in the thematic mapping (Fig 2). It was identified these could be organised into pairs, each with an antithesis. Findings are presented under each thematic pair.

#### 1. Cultural and family influence vs. cultural competency and diversity

The influence of culture impacted recruitment to studies. A woman’s culture influenced decisions when deciding whether to participate, and the extent to which cultural factors were considered in study design was key.

Cultural beliefs about the significance of the physical body influenced the decision to consent to a study. In communities ranging across African and Caribbean, Jewish and South Asian groups, the spiritual or religious meaning attached to the body meant if a study required sample collection they were less willing to participate, or declined that aspect [43, 44].

The influence of family in the decision to participate was seen particularly in Asian cultures, as women cited the opinion of a relative as a reason to decline participation, or even to withdraw consent. Often it was the view of the husband, however for some the mother-in-law was an influential factor [32, 44, 45].

Barriers were not restricted to cultural practices or beliefs, but also pragmatic reasons. If study materials were only available in the English language this was highlighted as a reason for lack of interest in trial participation [44, 45]. Multilingual resources appeared key to increasing recruitment. This included research staff who can recruit and perform follow-ups in the participant’s native language, and study materials e.g. invitation letters written in appropriate languages [44, 46, 47]. Additionally, using a targeted invitation letter for Latina women in the USA that was written in Spanish and acknowledged health disparities the ethnic group experienced increased participant screening rates [46].

Adapting communication to be culturally appropriate demonstrated respect, thus facilitating women’s relationships with both researchers and the research. This was particularly noted for women of South Asian origin [44]. One study which recruited Brazilians demonstrated understanding the cultural importance of friendliness and relating to people before getting “down to business” was facilitative to successful relationships [47].

Cultural competency was demonstrated in study designs which adapted processes to reflect an understanding of the norms, practices and beliefs of cultures. In a trial exploring postnatal depression it was acknowledged that for some ethnic minority groups mental health problems often had a stigma attached. The wording in the recruitment message was subsequently changed to focus on exploring health issues and stresses after childbirth, rather than using the term “depression” [48] which improved study acceptance. To ensure protocols and data collection instruments are linguistically and culturally appropriate the importance of including people on the research design team who are culturally congruent with the population was demonstrated [47]. A research team who shared the same cultural background with potential participants increased engagement and trust and therefore recruitment [44, 47, 49].

#### 2. Distrust vs. trust and community rapport

Lack of trust negatively impacted women’s willingness to participate in research. The concept of research participation being associated with being a “guinea pig” demonstrated underlying mistrust, particularly for Black British women [43].

Distrust was largely restricted to fears around data safety. Groups in the UK and USA demonstrated concerns about confidentiality and researchers sharing data. There was apprehension personal information gained from research could be shared with law enforcement agencies, immigration, and relatives or employers [43, 47, 48, 50]. Explicitly acknowledging concerns and giving open explanations about the ways data would be used and how it would remain confidential addressed this, thus increasing trust [48].

Trusting medical researchers increased the likelihood a woman would participate [50] and strategies to increase trust were key to successful recruitment. This was largely achieved through community outreach, enabling research staff to build relationships and develop rapport with potential participants. Consequently, there was increased trust in the research resulting in successful recruitment and retention [47, 49]. Involvement of community partners from the beginning of a research project was also integral to success. Endorsement and dissemination of information regarding the research by partners such as faith-based organisations and community leaders facilitated trust in the research for potential participants [47].

Outreach strategies included embedding researchers in social environments, including community centres, churches and social events. This built rapport and increased opportunities for information sharing and study recruitment [47, 49]. Successful outreach was strengthened by bilingual and culturally congenial research assistants [47, 49]. Personalising communication to reflect participant’s religious or cultural beliefs increased rapport and encouraged participant retention [44].

Developing rapport was not restricted to community outreach but was also successful through a regular presence of researchers in clinical areas. Familiarity with the research team was achieved through regular communication with staff and service users. Consequently, when potential participants were approached there was an existing rapport facilitating trust [49]. This had ongoing benefits throughout the study as participants developed relationships with research staff and felt valued, increasing retention [49]. Positive experiences of research and relationships with staff was important, as participants were likely to refer other potential participants. This personal recommendation increased trust in the research from the outset [47].

#### 3. Research design and demands vs. facilitative study design

The design of a study and how much it would demand from a woman was integral to how successful recruitment was.

Studies designed to recruit women from busy clinics found this was impeded by the lack of dedicated time and space for research, with regular interruptions and noisy environments reducing women’s willingness to participate [49]. Reflective and well managed recruitment strategies were key to successful recruitment. Maintaining records of which contact approaches yielded the most responses and pursuing these was effective. Additionally, assessing feedback from those who declined enabled strategies to be adjusted [47–49]. Direct recruitment methods involving personal contacts were more successful than indirect methods, such as social media posts, leaflets and posters in community centres [44, 47]. However, one trial found that although in-person recruitment was achieved higher screening rates, it did not always translate into enrolment. For one study advertisements in waiting areas were found to be the most successful approach in achieving a higher proportion of women who were screened and then successfully enrolled [51].

Well managed recruitment plans including successful communication strategies impacted participant recruitment and retention. Using multiple communication methods and undertaking several attempts to contact potential participants was effective. Subsequently, identifying participant’s preferences for ongoing communication and maintaining regular contact to remind them of follow-up visits was crucial [47, 49]. Offering participants a means of data collection which suited them aided successful delivery of a study, e.g. offering a choice of online, face-to-face, or telephone conducted questionnaires [48].

The time required to participate in research placed additional demands on women and was cited as a reason to decline participation. Women reported difficulty balancing work and family life alongside antenatal appointments, therefore, not wishing to contend with the additional perceived burden from research [43, 47, 48]. Using a convenient and flexible study design for women aimed to address this. Offering flexibility with study visits was conducive to success, providing participants with different location options, and weekend and evening appointments [47–49]. The option of combining study appointments with routine visits was also facilitative for some [43, 49]. However, for others, using a convenient and familiar location outside of the hospital was preferable; somewhere participants felt comfortable. These included local GP surgeries [43], churches, community centres, or their homes [47].

The procedures or information the research required was also an influential factor. Information taken from medical records was considered acceptable for most, however procedures considered unpleasant, including invasive swabs or blood tests, and infant or internal examinations were cited as barriers [43, 45, 50, 52]. Non-invasive tests were more favourable [43]. For studies using interviews or surveys it was important they were not lengthy [47, 49] and the option of completing it via different modes increased responses [48]. For an interventional study, preferring standard care was also a factor [32].

Women who were experiencing pregnancy related stress or complicated pregnancies did not feel they could take on the perceived burden of research. This was further impacted by the timing of approach if a woman was contending with emotional or physical responses to recent events [44, 45, 47]. A study design allowing time for women to consider participation, letting them lead further interactions, improved recruitment [48, 51].

#### 4. “What’s the Point?” vs. personal and community benefit

“What’s the Point?” captured the barrier of perceiving research to involve potential harm with no benefit. However, other women were more positive about research participation as they saw the potential for either personal benefit or benefit to their wider community.

The potential for harm was referred to in different formats and considered a risk in relation to physical procedures required by the study, potential psychological harm caused by participating, and harm that may result from sharing data [43, 48, 50]. For women who were asked about enrolling their unborn child into a birth cohort study, a strong consensus existed among different ethnic groups that they perceived the potential for harm as too significant when there was no direct benefit to participation, particularly if their child was healthy [43].

The offer of additional care, such as additional free antenatal ultrasound scans were cited as a reasons for participation, particularly for Black women in the USA [53]. More regular contact with healthcare providers was also seen as a benefit to research participation, assuming any potential ill health would be detected [43]. This perceived benefit was over-exaggerated and more prevalent in non-English speaking UK groups [43].

Women cited reasons for decline as lack of interest or not wanting to participate in any research [32, 44, 48], suggesting a lack of personal connection with research, or a feeling there were no benefits. Demonstrating the relevance of a study to the community was found to be facilitative for some. In one RCT, women who received an invitation letter that acknowledged disparities in the condition for their ethnicity, resulted in an improved screening rate [46]. However, this was specifically for Latina women who preferred to communicate in Spanish, and did not apply to those preferring English.

Personal benefits in the form of a material incentive encouraged participation [43, 47, 49]. Vouchers were preferable compared to cash incentives and were considered “more polite” for Jewish women [43]. Compensation was not associated with willingness to participate for all groups or research designs however [52, 54].

Although not explicitly stated by woman as a reason for participation, positive experiences of research likely contributed to the retention of participants. Women reported emotional support from their interactions with researchers [49]. Others felt there were educational benefits to participation, e.g. gaining knowledge about their pregnancy or the care of their baby [49, 53]. Conversely, for some women who had negative experiences and reported discrimination during pregnancy, this increased their willingness to participate in research [55].

Women demonstrated altruism, reporting reasons to participate as providing improvements in care and knowledge for all [43, 53, 55]. However, for some women, seeing a potential direct benefit for their family or cultural community was more important [43, 50]. This was also reflected in recruitment messages, whereby researchers who emphasised the potential impact of a study to inform better care for a woman’s own community found this was an important element in increasing participant investment [47, 48].

## Discussion

This review aimed to identify what has previously been found to influence ethnic minority women’s participation in maternity research. It has captured factors that influence a woman’s decision to participate in research and highlighted successful recruitment strategies. Whilst many of the reasons identified were highlighted in previous reviews which were not focused on ethnic minority women, findings around the impact of culture on decisions to participate and the cultural competency of research teams are unique to this population.

This review demonstrated clear barriers to participation included a lack of time, timing of approach and risk perception. These echo findings from different populations [33, 56]. The perception of risk in maternity research may carry extra weight, particularly in interventional studies as women must consider the risk to themselves and their unborn child. Perception of harm for a future child had key implications for decision-making [43, 50], similar to neonatal and paediatric research [57, 58]. Women in Garg et al’s study also highlighted being uncomfortable in making a decision on behalf of a child; a factor not reflected elsewhere [43]. For trials that require longer term follow-up of a child after birth this should be considered and explored further.

Most of the papers in the review were from the USA (10/13). Consequently, some findings may be less relevant to other settings due to differences in healthcare systems. In one interventional trial preferring standard care was a reason for decline, however this was less common for Black women and those without private healthcare [32]. Like the UK, Black women in the USA experience disparities in maternity care [59] and due to the nature of the US healthcare system it may have been perceived the invention was unobtainable unless participating in the research. Similarly, in another US study Black women more often reported participation due to concerns about pregnancy or the benefit of extra scans compared to White women [53].

Distrust is demonstrated in previous research as a key reason for hesitance to participate for those from ethnic minority communities [20, 28, 60–62], and for some White women approached for research in pregnancy [33, 63]. It was, therefore, surprising distrust was not expressed more frequently within this review, even when poor experiences of healthcare were reported [43]. This may reflect several of the study methodologies, whereby closed questions were used to determine reasons for research participating, or where reasons for decline in a study were documented without further exploration. More in-depth qualitative research is needed to gain further insights.

The use of culturally appropriate research materials and bilingual or culturally congruent researchers was reported as crucial to successful recruitment across several studies [44, 47, 49]. However, papers which reported using researchers of the same ethnic background as facilitative were based on findings inferred by the researchers, rather than reports from women. In studies examining women’s views this theme was not prominent. There may be several explanations for this: women may not be consciously aware of this influence; or women were not asked questions which would provide the opportunity to discuss this; or women do not place importance on this factor. Conversely, previous research in non-pregnant ethnic minority populations found having a researcher from the same cultural background was not always preferable, due to the fear of sharing information with someone who may be known to them, particularly when a condition is considered taboo [20]. Therefore, using cultural congruent researchers is not a solution for all potential participants or research questions.

Regardless of research staffs’ identities, community engagement and involvement remained a key theme in the papers which analysed recruitment strategies [47, 49], reflecting wider literature which cites this as key to successful recruitment of ethnic minority groups [20, 62, 64]. However, few studies have empirically tested the effectiveness of this approach [65] and no papers in this review tested their community-based recruitment approaches against other strategies.

Bilingual researchers and using study materials in other languages facilitated recruitment [44, 46, 47, 49], however outside of pregnancy it is highlighted that even without this, demonstrating respectful behaviour and active listening successfully builds trust [20]. The personal exchanges with researchers, and their communication skills were demonstrated in other studies to be influential in recruitment and retention to trials, often even more so than the information provided in study materials [56, 66–68]. Our review supported these findings, demonstrating the importance of developing rapport with potential participants. Due to reports of poor experiences with healthcare professional this may be even more significant in this population [69–71].

Cultural competence is a fundamental element in developing rapport and enabling understanding of the nuances of effective communication across different cultures. Cultural competency training for research staff has been identified as a successful strategy in improving inclusive recruitment [62]. Understanding differences between cultures may enable development of recruitment strategies. For example, in acknowledging the widely cited influence of family for South Asian women [32, 44, 45], strategies could be developed with women to explore how this could be addressed to reduce barriers.

Conducting research in a flexible way and accounting for personal preferences in methodologies was successful in increasing recruitment and maintaining retention [47–49]. A personalised approach to recruitment was highlighted as successful in previous reviews [62, 72], tailoring approaches in the same way that healthcare professionals should deliver individualised clinical care [63]. The personalised approach should apply even if a woman has previously taken part in research, as this does not necessarily correlate in increased trust or willingness to participate in future research [26, 61].

This review identified comparatively more facilitators compared to barriers which may be due to the study designs. Studies which analysed recruitment strategies focused on those that were successful, and papers that identified reasons for decline were often limited to unprompted responses from women without further exploration. This perhaps illustrates the paradox in attempting to gain a better understanding of why people may not want to participate in research; you are asking people to participate in research to investigate why they do not want to participate in research. To address this the importance of working with communities and developing trust is a vital first step in order to access the views of those who may be the most hesitant to get involved.

### Strengths and limitations

This is the first review to examine participation in maternity research for women from ethnic minority populations. Unlike previous reviews outside of pregnancy, this review was not restricted to clinical trials and therefore provided a broader perspective on the factors influencing participation in a range of research methodologies.

Included studies were mainly observational. Literature demonstrates when deciding to participate in a clinical trial of a medicinal product factors differ compared to observational research [66], therefore, the findings may not be applicable to these trials. Successful strategies to recruitment were illustrated, however the papers presenting these did not include participant’s views. Although results were presented as facilitative to recruitment they were largely based on the perceptions of the researchers, and were neither empirically tested nor necessarily supported by women’s views. Four studies which explored opinions from women related to a hypothetical trial. The disadvantage of this is one cannot determine whether these views would remain once presented with a real opportunity to participate.

Although there are clear areas for consideration in the design and delivery of a trial, barriers to inclusive research may be more systemic and run deeper than interactions between women and those who approach them. Evidence of structural racism exists throughout research processes, from design, funding, approvals and implementation [73, 74]. Without these elements first being addressed it may be very difficult to achieve truly inclusive research.

## Conclusions

This review highlighted the lack of in-depth rigorous research into factors influencing the decision to participate in maternity research for women from ethnic minority backgrounds. There was particularly a lack of representation of those from outside the USA.

Some of the main findings reflected the literature exploring research participation in the general population, however the importance of cultural competency in research design and delivery was a key factor and unique to those from ethnic minority groups. It is therefore recommended cultural competency training is available for those working in research.

Strategies improving the success of a study included community outreach, engagement and involvement. However, these were mainly employed when a specific ethnic group was being sampled; this may be more challenging when applied to larger clinical trials aiming for representation across several groups. Further research is required to explore how to facilitate diverse recruitment in large multi-centred trials within maternity.

Further research is needed to more accurately evaluate the multi-faceted influences affecting ethnically diverse recruitment to maternity research and to investigate the strategies that can facilitate this. There is a clear gap in findings from outside the USA and in the variety of methodologies used in maternity research. To ensure the success of further research involving service users from inception through to delivery is vital.

## Supporting information

S1 Checklist PRISMA 2009 checklist. (DOC)

S1 File MEDLINE search strategy. (DOC)

S2 File Mixed methods appraisal tool assessment. (DOCX)

## References

[1] Knight M. et al., ‘Saving Lives, Improving Mothers’ Care Maternal, Newborn and Infant Clinical Outcome Review *Programme’*, 2021, Accessed: Jan. 16, 2022. [Online]. Available: www.hqip.org.uk/national-programmes.

[2] Draper E. S. et al., ‘Maternal, Newborn and Infant Clinical Outcome Review Programme MBRRACE-UK Perinatal Mortality Surveillance Report’, 2021, Accessed: Jan. 16, 2022. [Online]. Available: www.hqip.org.uk/national-programmes.

[3] Public Health England, ‘Maternity high impact area: Reducing the inequality of outcomes for women from Black, Asian and Minority Ethnic (BAME) communities and their babies’, 2020. Accessed: May 08, 2021. [Online]. Available: https://assets.publishing.service.gov.uk/government/uploads/system/uploads/attachment_data/file/942480/Maternity_high_impact_area_6_Reducing_the_inequality_of_outcomes_for_women_from_Black__Asian_and_Minority_Ethnic__BAME__communities_and_their_babies.pdf

[4] Li Y., Quigley M. A., Macfarlane A., Jayaweera H., Kurinczuk J. J., and Hollowell J., ‘Ethnic differences in singleton preterm birth in England and Wales, 2006‐12: Analysis of national routinely collected data’, *Paediatr Perinat Epidemiol*, vol. 33, no. 6, pp. 449–458, Nov. 2019, doi: 10.1111/ppe.12585 31642102 PMC6900067

[5] NICE, ‘Diabetes in pregnancy’, 2015.

[6] Johnson J. D. and Louis J. M., ‘Does race or ethnicity play a role in the origin, pathophysiology, and outcomes of preeclampsia? An expert review of the literature’, *American Journal of Obstetrics and Gynecology*. Mosby Inc., Jul. 24, 2020. doi: 10.1016/j.ajog.2020.07.038 32717255

[7] Redshaw M. and Henderson J., ‘Who is actually asked about their mental health in pregnancy and the postnatal period? Findings from a national survey’, *BMC Psychiatry*, vol. 16, no. 1, pp. 1–8, Sep. 2016, doi: 10.1186/s12888-016-1029-9 27633660 PMC5025550

[8] Pilav S. et al., ‘A qualitative study of minority ethnic women’s experiences of access to and engagement with perinatal mental health care.’, *BMC Pregnancy Childbirth*, vol. 22, no. 1, p. 421, May 2022, doi: 10.1186/S12884-022-04698-9/TABLES/3 35585579 PMC9116695

[9] Knight M., Lewis G., Acosta C. D., and Kurinczuk J. J., ‘Maternal near-miss case reviews: the UK approach’, *BJOG*, vol. 121, no. Suppl 4, pp. 112–116, Sep. 2014, doi: 10.1111/1471-0528.12802 25236644 PMC4314674

[10] Knight M. et al., Saving Lives, Improving Mothers’ Care Maternal, Newborn and Infant Clinical Outcome Review Programme. 2020. Accessed: Apr. 07, 2021. [Online]. Available: www.hqip.org.uk/national-programmes.

[11] RCOG, ‘RCOG Position Statement: Racial disparities in women’s healthcare’, 2020.

[12] L. A. Penner, N. Hagiwara, S. Eggly, S. L. Gaertner, T. L. Albrecht, and J. F. Dovidio, ‘Racial healthcare disparities: A social psychological analysis’, 10.1080/10463283.2013.840973, vol. 24, no. 1, pp. 70–122, 2013. PMC4151477 25197206

[13] Bharj K. K. and Salway S. M., ‘Addressing ethnic inequalities in maternity service experiences and outcomes: responding to women’s needs and preferences’, 2008. Accessed: May 08, 2021. [Online]. Available: www.dh.gov.uk/en/Publicationsand

[14] Birthrights and Birth Companions, ‘Holding it all together: Understanding how far the human rights of woman facing disadvantages are respected during pregnancy, birth and postnatal care. ‘, 2019. https://www.birthrights.org.uk/wp-content/uploads/2019/09/Holding-it-all-together-Full-report-FINAL-Action-Plan.pdf (accessed May 09, 2021).

[15] Kapadia D. et al., ‘Ethnic Inequalities in Healthcare: A Rapid Evidence Review’, 2022.

[16] Awe T., Abe C., Peter M., and Wheeler R., ‘THE BLACK MATERNITY EXPERIENCES SURVEY A NATIONWIDE STUDY OF BLACK WOMEN’S EXPERIENCES OF MATERNITY SERVICES IN THE UNITED KINGDOM’, 2022.

[17] Birthrights, ‘Systemic racism, not broken bodies. An inquiry into racial injustice and human rights in UK maternity care’, 2022.

[18] Liu B., Nadeem U., | Alexander Frick, Alakaloko M., | Amar Bhide, and Thilaganathan B., ‘Reducing health inequality in Black, Asian and other minority ethnic pregnant women: impact of first trimester combined screening for placental dysfunction on perinatal mortality’, *BJOG*, vol. 00, pp. 1–7, Feb. 2022, doi: 10.1111/1471-0528.17109 35104381 PMC9544950

[19] RCM, ‘Race matters’, 2020. https://www.rcm.org.uk/supporting/race-matters/ (accessed Apr. 10, 2021).

[20] Farooqi A. et al., ‘Developing a toolkit for increasing the participation of black, Asian and minority ethnic communities in health and social care research’, *BMC Med Res Methodol*, vol. 22, no. 1, pp. 1–16, Dec. 2022, doi: 10.1186/S12874-021-01489-2/TABLES/3 35026996 PMC8758375

[21] Robertson R., Williams E., Buck D., and Breckwoldt J., ‘Ethnic health inequalities and the NHS Driving progress in a changing system’, 2021. Accessed: Jun. 06, 2022. [Online]. Available: www.kingsfund.org.uk

[22] Chen M. S., Lara P. N., Dang J. H. T., Paterniti D. A., and Kelly K., ‘Twenty years post-NIH Revitalization Act: Enhancing minority participation in clinical trials (EMPaCT): Laying the groundwork for improving minority clinical trial accrual’, *Cancer*, vol. 120, pp. 1091–1096, 2014, doi: 10.1002/CNCR.28575 24643646 PMC3980490

[23] Khunti K. et al., ‘Representation of people of South Asian origin in cardiovascular outcome trials of glucose-lowering therapies in Type 2 diabetes’, *Diabetic Medicine*, vol. 34, no. 1, pp. 64–68, Jan. 2017, doi: 10.1111/DME.13103 26926478

[24] VH M., HM K., and CP G., ‘Participation in cancer clinical trials: race-, sex-, and age-based disparities’, *JAMA*, vol. 291, no. 22, pp. 2720–2726, Jun. 2004, doi: 10.1001/jama.291.22.2720 15187053

[25] Diehl K. M. et al., ‘Features Associated with Successful Recruitment of Diverse Patients onto Cancer Clinical Trials: Report from the American College of Surgeons Oncology Group’, *Oncol*, vol. 18, pp. 3544–3550, 2011, doi: 10.1245/s10434-011-1818-9 21681382 PMC5773065

[26] Smart A. and Harrison E., ‘The under-representation of minority ethnic groups in UK medical research’, *Ethn Health*, vol. 22, no. 1, pp. 65–82, Jan. 2017, doi: 10.1080/13557858.2016.1182126 27174778

[27] Caplan A. and Friesen P., ‘Health disparities and clinical trial recruitment: Is there a duty to tweet?’, *PLoS Biol*, vol. 15, no. 3, p. e2002040, Mar. 2017, doi: 10.1371/journal.pbio.2002040 28249024 PMC5331960

[28] Clark L. T. et al., ‘Increasing Diversity in Clinical Trials: Overcoming Critical Barriers’, *Current Problems in Cardiology*, vol. 44, no. 5. Mosby Inc., pp. 148–172, May 01, 2019. doi: 10.1016/j.cpcardiol.2018.11.002 30545650

[29] Commission on Race and Ethnic Disparities (CRED), ‘Commission on Race and Ethnic Disparities: The Report–March 2021’, 2021.

[30] Healy P. et al., ‘Identifying trial recruitment uncertainties using a James Lind Alliance Priority Setting Partnership—the PRioRiTy (Prioritising Recruitment in Randomised Trials) study’, *Trials*, vol. 19, no. 1, Mar. 2018, doi: 10.1186/s13063-018-2544-4 29490702 PMC5831203

[31] Treweek S. et al., ‘Developing the INCLUDE Ethnicity Framework—a tool to help trialists design trials that better reflect the communities they serve’, *Trials*, vol. 22, no. 1, pp. 1–12, Dec. 2021, doi: 10.1186/S13063-021-05276-8/TABLES/3 33971916 PMC8108025

[32] Mallett G. et al., ‘Characteristics Associated With Consent and Reasons for Declining in a Randomized Trial in Pregnancy’, *Obstetrics and gynecology*, vol. 136, no. 4, pp. 731–737, Oct. 2020, doi: 10.1097/AOG.0000000000003998 32925629 PMC7971102

[33] van der Zande I. S. E., van der Graaf R., Hooft L., and van Delden J. J. M., ‘Facilitators and barriers to pregnant women’s participation in research: A systematic review’, *Women and Birth*, vol. 31, no. 5. Elsevier B.V., pp. 350–361, Oct. 01, 2018. doi: 10.1016/j.wombi.2017.12.009 29373261

[34] Stern C. et al., ‘Methodological guidance for the conduct of mixed methods systematic reviews’, *JBI Evid Synth*, vol. 18, no. 10, pp. 2108–2118, Oct. 2020, doi: 10.11124/JBISRIR-D-19-00169 32813460

[35] Sandelowski M., Voils C. I., and Barroso J., ‘Defining and Designing Mixed Research Synthesis Studies’, 2006. PMC2809982 20098638

[36] Joanna Briggs Institute, ‘Methodology for JBI Mixed Methods Systematic Reviews’, 2014. Accessed: Jun. 17, 2021. [Online]. Available: www.joannabriggs.org

[37] Hong Q. N., Pluye P., Bujold M., and Wassef M., ‘Convergent and sequential synthesis designs: implications for conducting and reporting systematic reviews of qualitative and quantitative evidence’, *Systematic reviews*, vol. 6, no. 1. BioMed Central, p. 61, Mar. 23, 2017. doi: 10.1186/s13643-017-0454-2 28335799 PMC5364694

[38] Thomas J. and Harden A., ‘Methods for the thematic synthesis of qualitative research in systematic reviews’, *BMC Med Res Methodol*, vol. 8, p. 45, 2008, doi: 10.1186/1471-2288-8-45 18616818 PMC2478656

[39] QSR International Pty Ltd., ‘NVivo (released in March 2020)’, 2020. https://www.qsrinternational.com/nvivo-qualitative-data-analysis-software/home (accessed Mar. 02, 2022).

[40] Pope C., Mays N., and Popay J., Synthesizing Qualitative and Quantitative Health Research. Adelaide: Ramsay, 2007. Accessed: Jun. 10, 2021. [Online]. Available: https://ebookcentral.proquest.com/lib/kcl/detail.action?docID=316315

[41] Nha HONG Q. et al., ‘MIXED METHODS APPRAISAL TOOL (MMAT) VERSION 2018 User guide’. Canadian Intellectual Property Office, Industry Canada, 2018. Accessed: May 02, 2021. [Online]. Available: http://mixedmethodsappraisaltoolpublic.pbworks.com/

[42] Page M. J. et al., ‘The PRISMA 2020 statement: An updated guideline for reporting systematic reviews’, *The BMJ*, vol. 372. BMJ Publishing Group, Mar. 29, 2021. doi: 10.1136/bmj.n71 33782057 PMC8005924

[43] Garg N., Round T. P., Daker-White G., Bower P., and Griffiths C. J., ‘Attitudes to participating in a birth cohort study, views from a multiethnic population: a qualitative study using focus groups’, *Health Expectations*, vol. 20, no. 1, pp. 146–158, Feb. 2017, doi: 10.1111/hex.12445 27312575 PMC5217869

[44] Neelotpol S., Hay A. W. M., Jolly A. J., and Woolridge M. W., ‘Challenges in collecting clinical samples for research from pregnant women of South Asian origin: evidence from a UK study’, *BMJ Open*, vol. 6, p. 10554, 2016, doi: 10.1136/bmjopen-2015-010554 27580825 PMC5013462

[45] van Delft K., Schwertner-Tiepelmann N., Thakar R., and Sultan A. H., ‘Recruitment of pregnant women in research’, *J Obstet Gynaecol (Lahore)*, vol. 33, no. 5, pp. 442–446, Jul. 2013, doi: 10.3109/01443615.2013.767787 23815192

[46] Brown S. D. et al., ‘Outreach to diversify clinical trial participation: A randomized recruitment study’, *Clinical Trials*, vol. 12, no. 3, pp. 205–211, Jun. 2015, doi: 10.1177/1740774514568125 25644997 PMC4424096

[47] Cristina Lindsay A. et al., ‘Faith, Family, and Social Networks: Effective Strategies for Recruiting Brazilian Immigrants in Maternal and Child Health Research’, *J Racial Ethn Health Disparities*, vol. 8, pp. 47–59, 2021, doi: 10.1007/s40615-020-00753-3 32458344

[48] Martin A., Negron R., Balbierz A., Bickell N., and Howell E. A., ‘Recruitment of Black and Latina Women to a Randomized Controlled Trial’, *J Health Care Poor Underserved*, vol. 24, no. 3, pp. 1102–1114, 2013, doi: 10.1353/hpu.2013.0125 23974384

[49] Barnett J., Aguilar S., Brittner M., and Bonuck K., ‘Recruiting and retaining low-income, multi-ethnic women into randomized controlled trials: Successful strategies and staffing’, *Contemp Clin Trials*, vol. 33, no. 5, pp. 925–932, Sep. 2012, doi: 10.1016/j.cct.2012.06.005 22732312 PMC3430447

[50] Savich R. D., Tigges B. B., Rios L. I., McCloskey J., Tollestrup K., and Annett R. D., ‘Willingness of women to participate in obstetrical and pediatric research involving biobanks’, *J Community Genet*, vol. 11, no. 2, pp. 215–223, Apr. 2020, doi: 10.1007/s12687-019-00446-3 31782046 PMC7062944

[51] Gillespie S. L., ‘A Comparison of Recruitment Methods for a Prospective Cohort Study of Perinatal Psychoneuroimmunology among Black American Women’, *J Urban Health*, vol. 98, no. Suppl 2, pp. 115–122, Oct. 2021, doi: 10.1007/s11524-021-00548-9 34152521 PMC8501172

[52] Nechuta S., Mudd L. M., Biery L., Elliott M. R., Lepkowski J. M., and Paneth N., ‘Attitudes of pregnant women towards participation in perinatal epidemiological research’, *Paediatr Perinat Epidemiol*, vol. 23, no. 5, pp. 424–430, Sep. 2009, doi: 10.1111/j.1365-3016.2009.01058.x 19689493

[53] Lamvu G., Lorenz C., Funk M. J., Makarushka C., Hartmann K., and Savitz D., ‘Racial differences among reasons for participating in research of pregnancy outcomes: The right from the start experience’, *Gend Med*, vol. 2, no. 3, pp. 166–173, Sep. 2005, doi: 10.1016/s1550-8579(05)80045-2 16290889

[54] Nechuta S., Mudd L. M., Elliott M. R., Lepkowski J. M., and Paneth N., ‘Attitudes of pregnant women towards collection of biological specimens during pregnancy and at birth’, *Paediatr Perinat Epidemiol*, vol. 26, no. 3, pp. 272–275, May 2012, doi: 10.1111/j.1365-3016.2012.01265.x 22471686

[55] Gatny H. H. and Axinn W. G., ‘Willingness to Participate in Research during Pregnancy: Race, Experience, and Motivation’, *Field Methods*, vol. 24, no. 2. NIH Public Access, pp. 135–154, May 2012. doi: 10.1177/1525822X11419819 22798727 PMC3393046

[56] Houghton C. et al., ‘Factors that impact on recruitment to randomised trials in health care: a qualitative evidence synthesis’, *Cochrane Database Syst Rev*, vol. 2020, no. 10, Oct. 2020, doi: 10.1002/14651858.MR000045.PUB2 33026107 PMC8078544

[57] McKechnie L. and Gill A. B., ‘Consent for neonatal research’, *Arch Dis Child Fetal Neonatal Ed*, vol. 91, no. 5, p. F374, Sep. 2006, doi: 10.1136/adc.2005.075036 16923938 PMC2672847

[58] Hoberman A. et al., ‘Factors That Influence Parental Decisions to Participate in Clinical Research: Consenters vs Nonconsenters’, *JAMA Pediatr*, vol. 167, no. 6, pp. 561–566, Jun. 2013, doi: 10.1001/JAMAPEDIATRICS.2013.1050 23546617 PMC3674159

[59] H. Yoder and L. R. Hardy, ‘Midwifery and Antenatal Care for Black Women: A Narrative Review’:, 10.1177/2158244017752220, vol. 8, no. 1, Jan. 2018.

[60] Corbie-Smith G., Thomas S. B., and st D. M. M. George, ‘Distrust, race, and research’, *Arch Intern Med*, vol. 162, no. 21, pp. 2458–2463, Nov. 2002, doi: 10.1001/ARCHINTE.162.21.2458 12437405

[61] Scharf D. P., Mathews K. J., Jackson P., Hofsuemmer J., Martin E., and Edwards D., ‘More than Tuskegee: Understanding Mistrust about Research Participation’, *J Health Care Poor Underserved*, vol. 21, no. 3, pp. 879–897, Aug. 2010, doi: 10.1353/hpu.0.0323 20693733 PMC4354806

[62] Bodicoat D. H. et al., ‘Promoting inclusion in clinical trials—a rapid review of the literature and recommendations for action’, *Trials*, vol. 22, no. 1, pp. 1–11, Dec. 2021, doi: 10.1186/S13063-021-05849-7/TABLES/1 34863265 PMC8643184

[63] Baker L., Lavender T., and Tincello D., ‘Factors that influence women’s decisions about whether to participate in research: an exploratory study’, *Birth*, vol. 32, no. 1, pp. 60–66, Mar. 2005, doi: 10.1111/J.0730-7659.2005.00346.X 15725206

[64] National Institute for Health Research, ‘Improving inclusion of under-served groups in clinical research: Guidance from INCLUDE project’, 2020. https://www.nihr.ac.uk/documents/improving-inclusion-of-under-served-groups-in-clinical-research-guidance-from-include-project/25435 (accessed Jan. 16, 2022).

[65] Nicholson L. M., Schwirian P. M., and Groner J. A., ‘Recruitment and retention strategies in clinical studies with low-income and minority populations: Progress from 2004–2014’, *Contemporary Clinical Trials*, vol. 45. Elsevier Inc., pp. 34–40, May 01, 2015. doi: 10.1016/j.cct.2015.07.008 26188163

[66] Kenyon S., Dixon-Woods M., Jackson C. J., Windridge K., and Pitchforth E., ‘Participating in a trial in a critical situation: a qualitative study in pregnancy’, *Qual Saf Health Care*, vol. 15, no. 2, p. 98, Apr. 2006, doi: 10.1136/qshc.2005.015636 16585108 PMC2464828

[67] Myles S. et al., ‘A Multicenter Investigation of Factors Influencing Women’s Participation in Clinical Trials’, *J Womens Health*, vol. 27, no. 3, pp. 258–270, Mar. 2018, doi: 10.1089/jwh.2017.6458 29148879

[68] Tooher R. L., Middleton P. F., and Crowther C. A., ‘A thematic analysis of factors influencing recruitment to maternal and perinatal trials’, *BMC Pregnancy and Childbirth*, vol. 8, no. 1. BioMed Central, p. 36, Aug. 07, 2008. doi: 10.1186/1471-2393-8-36 18687110 PMC2532678

[69] J. Jomeen and M. Redshaw, ‘Ethnic minority women’s experience of maternity services in England’, 10.1080/13557858.2012.730608, vol. 18, no. 3, pp. 280–296, Jun. 2013. 23039872

[70] Henderson J., Gao H., and Redshaw M., ‘Experiencing maternity care: The care received and perceptions of women from different ethnic groups’, *BMC Pregnancy Childbirth*, vol. 13, no. 1, pp. 1–14, Oct. 2013, doi: 10.1186/1471-2393-13-196/TABLES/8 24148317 PMC3854085

[71] John J. R., Curry G., and Cunningham-Burley S., ‘Exploring ethnic minority women’s experiences of maternity care during the SARS-CoV-2 pandemic: a qualitative study’, *BMJ Open*, vol. 0, p. 50666, 2021, doi: 10.1136/bmjopen-2021-050666 34489290 PMC8423508

[72] Treweek S. et al., ‘Strategies to improve recruitment to randomised trials’, *Cochrane Database Syst Rev*, vol. 2018, no. 2, Feb. 2018, doi: 10.1002/14651858.MR000013.pub6 29468635 PMC7078793

[73] Powell R. A. et al., ‘Tackling racism in UK health research’, *BMJ*, vol. 376, Jan. 2022, doi: 10.1136/bmj-2021-065574 35042720 PMC8764577

[74] Carter E. B. and Mazzoni S. E., ‘A paradigm shift to address racial inequities in perinatal healthcare’, *Am J Obstet Gynecol*, vol. 224, no. 4, pp. 359–361, Apr. 2021, doi: 10.1016/j.ajog.2020.11.040 33306974

