# Correction: Factors which influence ethnic minority women’s participation in maternity research: A systematic review of quantitative and qualitative studies

**DOI:** 10.1371/journal.pone.0319820

**Published:** 2025-02-19

**Authors:** The PLOS One Staff

An incorrect version of Fig 2 was published in error. Please see the complete Fig 2 here.

**Fig 2:**
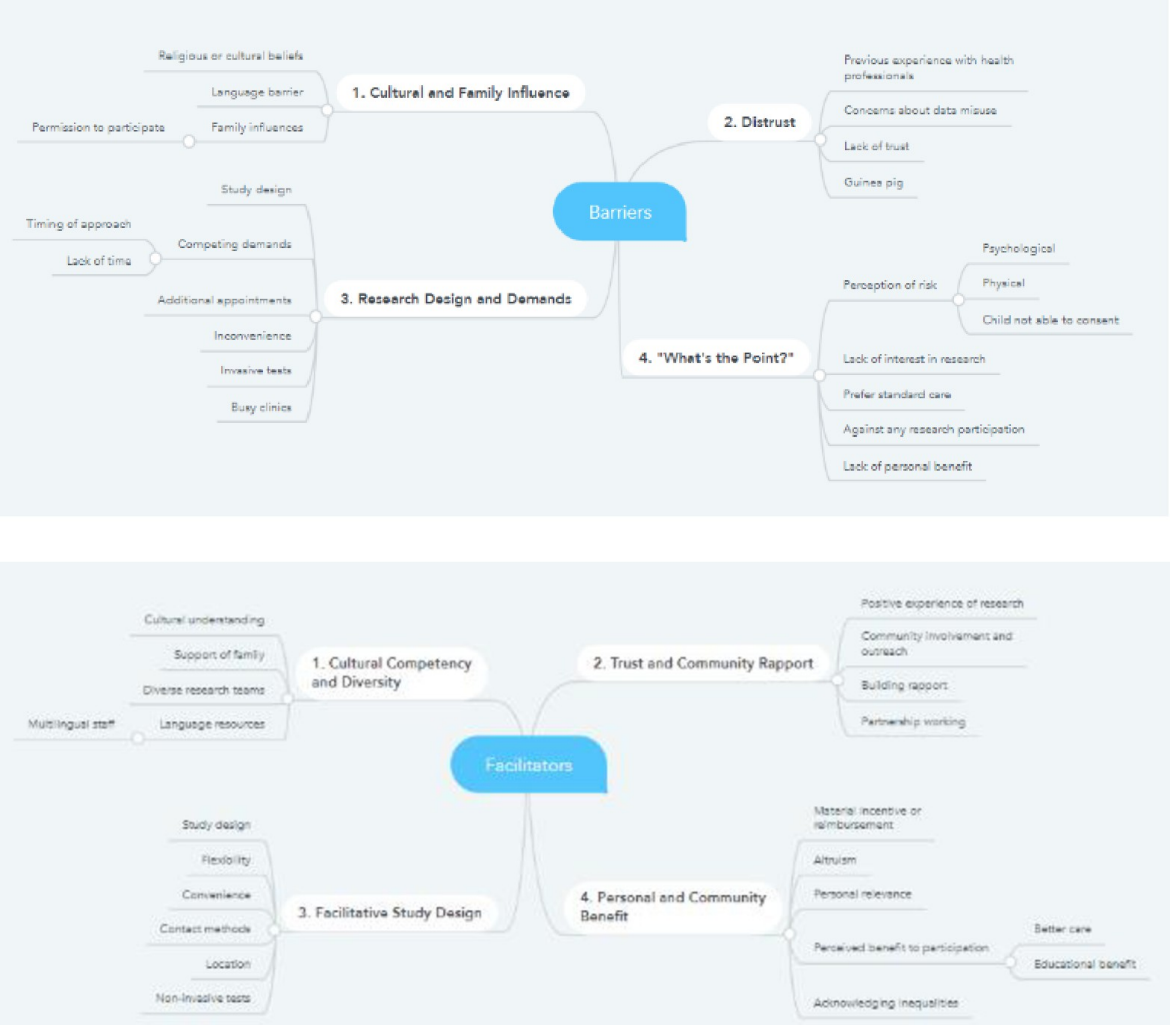
Thematic Maps of Barriers and Facilitators.
