# Supplementary material for: Factors which influence ethnic minority women’s participation in maternity research: A systematic review of quantitative and qualitative studies
Source: PLoS One. 2023 Feb 24;18(2):e0282088. doi: 10.1371/journal.pone.0282088 (PMC9956875; doi:10.1371/journal.pone.0282088)
Supplement: S2 File — (DOCX) [file pone.0282088.s003.docx]

**S1. Appendix: Mixed Methods Appraisal Tool Assessment**

| **Author**  **(year)** | **Qualitative** | |  |  |  | **Quantitative. Randomized Control Trial** | | | |  | **Comments** |
| --- | --- | --- | --- | --- | --- | --- | --- | --- | --- | --- | --- |
|  | 1.1. | 1.2. | 1.3. | 1.4. | 1.5. | 2.1. | 2.2. | 2.3. | 2.4. | 2.5. |  |
| Garg et al (2016) | **✓** | **✓** | **✓** | **✓** | **✓** |  |  |  |  |  |  |
| Brown et al (2015) |  |  |  |  |  | **✓** | **✓** | **✓** | **✓** | ? | It could not be determined whether participants read the invitation letter which was the intervention. |
|  |  |  |  |  |  |  |  |  |  |  |  |
|  | **Quantitative descriptive** | | |  |  | **Mixed methods** | |  |  |  | **Comments** |
|  | 4.1. | 4.2. | 4.3. | 4.4. | 4.5. | 5.1. | 5.2. | 5.3. | 5.4. | 5.5. |  |
| Mallet et al (2020) | **✓** | ? | **✓** | **✓** | **✓** |  |  |  |  |  | Some sites did not allow staff to ask about reasons for decline, therefore inconsistency in data collection. |
| Neelotpol et al (2016) | **✓** | **✓** | X | **✓** | X |  |  |  |  |  | Motivation to participate judged by the researchers’ perception, not a validated or reliable measure. |
| van Delft et al (2013) | **✓** | **✓** | **✓** | **✓** | **✓** |  |  |  |  |  | Would have been beneficial to also include an analysis exploring differences between groups. |
| Nechuta et al (2012) | ? | ? | **✓** | X | **✓** |  |  |  |  |  | A large proportion of missing data is noted. |
| Nechuta et al (2009) | **✓** | ? | **✓** | **✓** | **✓** |  |  |  |  |  |  |
| Lamvu et al (2005) | **✓** | ? | **✓** | **✓** | **✓** |  |  |  |  |  |  |
| Gatny and Axinn (2011) | **✓** | ? | **✓** | ? | **✓** |  |  |  |  |  | There is not information given regarding missing data. Interpretation of the statistics appears correct as far as can be assessed. |
| Gillespie (2021) | **✓** | **✓** | **✓** | **✓** | **✓** |  |  |  |  |  |  |
| Lindsay et al (2021) |  |  |  |  |  | X | X | **✓** | ? | X | The data that were analysed for the qualitative component could not be guaranteed to be complete, as it was not originally collected for data collection purposes. |
| Barnett et al (2012) |  |  |  |  |  | X | **✓** | **✓** | X | **✓** | Although mixed methods are used, there is no discussion regarding the methodology. |
| Savich et al (2020) |  |  |  |  |  | **✓** | X | X | X | X | Focus groups discussions are not included in the findings, only the quantitative elements are presented. |
| Martin et al (2013) |  |  |  |  |  | X | **✓** | **✓** | ? | ? | The paper is not described as mixed methods; however this is the closest fit. It is therefore difficult to accurately assess the quality criterion. |

**Qualitative**

1.1. Is the qualitative approach appropriate to answer the research question?

1.2. Are the qualitative data collection methods adequate to address the research question?

1.3. Are the findings adequately derived from the data?

1.4. Is the interpretation of results sufficiently substantiated by data?

1.5. Is there coherence between qualitative data sources, collection, analysis and interpretation?

**Quantitative Randomised Controlled Trial**

2.1. Is randomization appropriately performed?

2.2. Are the groups comparable at baseline?

2.3. Are there complete outcome data?

2.4. Are outcome assessors blinded to the intervention provided?

2.5 Did the participants adhere to the assigned intervention?

**Quantitative Descriptive**

4.1. Is the sampling strategy relevant to address the research question?

4.2. Is the sample representative of the target population?

4.3. Are the measurements appropriate?

4.4. Is the risk of nonresponse bias low?

4.5. Is the statistical analysis appropriate to answer the research question?

**Mixed Methods**

5.1. Is there an adequate rationale for using a mixed methods design to address the research question?

5.2. Are the different components of the study effectively integrated to answer the research question?

5.3. Are the outputs of the integration of qualitative and quantitative components adequately interpreted?

5.4. Are divergences and inconsistencies between quantitative and qualitative results adequately addressed?

5.5. Do the different components of the study adhere to the quality criteria of each tradition of the methods involved?
